# Clinical Outcomes and Prognostic Factors for Patients with Malignant Peripheral Nerve Sheath Tumour

**DOI:** 10.1155/2021/8335290

**Published:** 2021-11-24

**Authors:** Yoshinori Imura, Hidetatsu Outani, Satoshi Takenaka, Naohiro Yasuda, Sho Nakai, Takaaki Nakai, Toru Wakamatsu, Hironari Tamiya, Kenichiro Hamada, Shigeki Kakunaga

**Affiliations:** ^1^Department of Orthopedic Surgery, Osaka University Graduate School of Medicine, 2-2 Yamadaoka, Suita 565-0871, Osaka, Japan; ^2^Musculoskeletal Oncology Service, Osaka International Cancer Institute, 3-1-69 Otemae, Chuo-ku, Osaka 541-8567, Japan; ^3^Department of Orthopedic Surgery, Osaka National Hospital, 2-1-14 Hoenzaka, Chuo-ku, Osaka 540-0006, Japan

## Abstract

**Introduction:**

Few studies have described the characteristics and prognostic factors of patients with malignant peripheral nerve sheath tumour (MPNST). In this study, we retrospectively investigated the clinicopathological features, clinical outcomes, and prognostic factors of these patients. *Patients and Methods*. We recruited patients with MPNST who were treated at our institutions from 1991 to 2020. We collected and statistically analysed information on patient-, tumour-, and treatment-related factors. The median follow-up period was 61 months (range, 1–335.8 months).

**Results:**

A total of 60 patients (31 males, 29 females) with a median age of 55 years (range, 8–84 years) at initial diagnosis were included. The median tumour size was 7 cm (range, 1.6–30 cm) in the greatest dimension. The 5-year overall survival (OS) rate of all patients was 69.5%. Univariate analysis revealed that large-sized tumour, metastasis at diagnosis, and no surgery of the primary tumour were significantly associated with patients with worse OS. Multivariate analysis identified surgery of the primary tumour as an independent prognostic factor for improved OS. Among patients with localised disease at diagnosis who underwent surgery of the primary tumour at our institutions, the 5-year OS, local recurrence-free survival (LRFS), and metastasis-free survival (MFS) rates were 81.1%, 78.2%, and 70.3%, respectively. Univariate analysis revealed that positive surgical margin was significantly correlated with unfavourable OS and LRFS, and high grade was a poor prognostic indicator for MFS.

**Conclusion:**

Complete surgical resection with negative surgical margins is necessary for a successful MPNST treatment. Multidisciplinary management of MPNST with aggressive features is important for optimising patient outcomes.

## 1. Introduction

Malignant peripheral nerve sheath tumour (MPNST) is highly malignant and accounts for approximately 5%–10% of all soft tissue sarcomas [1–10]. It typically occurs in patients between the ages of 20 and 50 years [1–3, 9]. Pathologically, MPNST is derived from the peripheral nerve and shows nerve sheath malignant differentiation [4, 9]. Usually, it clinically presents as an enlarging soft tissue mass emerging in the trunk, extremities, or head and neck region, with or without pain and dysesthesia.

Malignant lesions occur either sporadically or in association with neurofibromatosis Type 1 (NF1). NF1 results from a gain-of-function mutation in the *NF* gene and is inherited in an autosomal dominant fashion. Whereas earlier studies have estimated that NF1 patients' lifetime risk of developing MPNST is 1%–2%, more recent analyses have estimated the risk to be 8%–13% [3, 11, 12]. Moreover, NF1 may be underdiagnosed or unrecognised; ultimately, 20%–50% of patients with MPNST have been found to have NF1 [5, 9, 11–14].

MPNST may behave aggressively, with a high rate of local recurrence and distant metastasis [13, 15, 16]. Despite multidisciplinary therapy, the prognosis of MPNST is poor, with 5-year survival rates between 30% and 60% [3, 9, 12–23]. To date, surgery is the only proven therapy that increases survival in localised MPNST [13, 16].

The dismal outcome points to the urgent need to establish better therapeutic strategies for patients with MPNST and highlights the importance of identifying clinicopathological factors that affect prognosis. However, information obtained from a large cohort of Japanese patients with MPNST is lacking.

This retrospective study aimed to investigate the clinicopathological features, clinical outcomes, and prognostic factors in patients with MPNST treated at our affiliated hospitals.

## 2. Materials and Methods

We designed a multi-institutional retrospective study that was conducted in our institutions. We reviewed the records of each institute between January 1991 and June 2020. The patient eligibility criteria included MPNST diagnosis, as pathologically confirmed by an expert musculoskeletal tumour pathologist at each institute. MPNST usually consists of spindle cells demonstrating nuclear atypia, increased mitotic activity, and geographic areas of necrosis. MPNST demonstrates variable expression of immunohistochemical neural markers such as S100 and SOX10. All cases were diagnosed as MPNST. This study was approved by the Institutional Review Boards of each institution.

A total of 60 patients with MPNST who were treated at our hospitals were included in this study. Information on patient-related factors (age, sex, and NF1 status), tumour-related factors (site of primary lesions; tumour size, depth, and histological grade; and presence or absence of metastasis at initial diagnosis), treatment-related factors (surgery of the primary tumour and metastatic lesions, tumour surgical margin, and chemotherapy and radiotherapy status), local and distant relapse, follow-up period, and oncological outcome at final follow-up were anonymously collected from the medical charts of the patients. We were unable to obtain data on tumour size, histological grade, and surgical margin in three patients who received their first surgery at other hospitals. Data on tumour depth could not be collected in one patient.

We calculated the overall survival (OS) from the date of diagnosis to the date of death from any cause or the last follow-up visit. In patients who underwent surgery, we calculated the local recurrence-free survival (LRFS) from the date of surgery to the date of local recurrence or the last follow-up. In patients without distant metastasis at presentation, we calculated the metastasis-free survival (MFS) from the date of diagnosis to the date of metastasis or the last follow-up. Patients without surgery of the primary lesion and with metastasis at initial referral were excluded from the LRFS and MFS analysis, respectively. We calculated the OS, LRFS, and MFS using the Kaplan–Meier method and evaluated the impact of prognostic factors using the log-rank test in a univariate analysis.

We conducted a multivariate analysis using the Cox proportional hazards model, with variables chosen by using a forward conditional stepwise approach. Differences were considered significant when *p* values were <0.05. The EZR software (Saitama Medical Center, Jichi Medical University, Saitama, Japan), a graphical user interface for *R* (The *R* Foundation for Statistical Computing, Vienna, Austria), was used in the statistical analyses.

## 3. Results

### 3.1. Patient-, Tumour-, and Treatment-Related Characteristics

The median follow-up period was 61.2 months (range, 1–335.8 months) for all patients. The patient-, tumour-, and treatment-related characteristics of the 60 cases are presented in Table 1. The 31 male (51.7%) and 29 female (48.3%) patients had a median age of 55 years (range, 8–84 years) at initial diagnosis. A total of 27 patients (45%) were ≤50 years of age, and 33 patients (55%) were >50 years. Thirty-two patients (53.3%) had tumours that were related to NF1, and 28 (46.7%) had sporadic tumours.

The sites of primary lesions were the extremities in 19 patients (31.7%), the trunk in 35 (58.3%), and the head and neck in 6 (10%). The tumour size was ≤5 cm in the greatest dimension in 24 patients (42.1%) and >5 cm in 33 patients (57.9%), with a median size of 7 cm (range, 1.6–30 cm). The tumour depth was categorised as either superficial or deep in the investing fascia. A total of 20 patients (33.9%) had superficial tumours, and 39 (66.1%) had deep tumours.

Using the Fédération Nationale des Centres de Lutte Contre le Cancer (FNCLCC) grading system, we determined the histological grade [24]. FNCLCC Grade 1 tumours were considered low grade, whereas FNCLCC Grade 2 or 3 tumours were considered high grade. Twelve patients (21.1%) had low-grade tumours, and 45 (78.9%) had high-grade tumours. Fifty-five patients (91.7%) had localised disease, and five (8.3%) had metastatic disease at initial diagnosis.

Fifty-two patients (86.7%) underwent surgery for the primary tumour. The surgical margin was described as negative when the pathologist found no tumour cells at the edge of the material removed. In contrast, the margin was described as positive when tumour cells were found at the outer edge. Among them, a negative surgical margin was achieved in 36 patients, a positive margin was noted in 13 patients, and a surgical margin could not be obtained in 3 patients. The remaining eight patients (13.3%) could not undergo surgery due to inoperative local conditions for surgical treatment. Among these, three patients with localised disease who were judged as medically inoperable received carbon ion radiotherapy, and five patients received palliative chemotherapy and/or radiotherapy at the primary tumour site.

Chemotherapy and radiotherapy were given to 27 (45%) and 24 (40%) patients, respectively. Various chemotherapy regimens, including doxorubicin (DXR), ifosfamide (IFM), DXR/IFM, gemcitabine/docetaxel, trabectedin, eribulin, and pazopanib, were administered. DXR/IFM was the most commonly used regimen and demonstrated an objective response rate (ORR) of 27.3% and a disease control rate (DCR) of 81.8%. Among the 50 patients who had no metastasis at initial diagnosis and received surgery of their primary tumour, 11 and 8 patients received neoadjuvant and/or adjuvant chemotherapy and radiotherapy, respectively.

### 3.2. Survival and Outcomes

At the final follow-up, 26 patients (43.3%) were continuously disease-free, 5 (8.3%) had no evidence of disease, 3 (5%) were alive with the disease, 23 (38.3%) were dead from the disease, and 3 (5%) had died from other causes. The 5-year OS rate of all patients was 69.5%, with a median OS period of 132.5 months (range, 1–335.8 months).

Among the 55 patients with localised disease, distant metastasis occurred in 20 patients during follow-up, with a median MFS duration of 22.7 months (range, 3–189.7 months). The most common metastatic sites were the lungs, followed by bone, lymph nodes, and liver. Among the 52 patients who underwent surgery of the primary tumour, local recurrence developed in 17, with a median LRFS period of 12.9 months (range, 2.4–120.4 months). In 16 patients, surgical removal of the local recurrent tumour was performed.

A total of 50 patients (83.3%) had no distant metastasis at initial diagnosis and underwent surgery of the primary tumour. Among them, eight who received unplanned surgery at other hospitals were referred to our institutions after developing local recurrence and then underwent surgery of the recurrent tumour. Therefore, the remaining 42 patients with localised disease at diagnosis underwent surgery of the primary tumour at our hospitals. Among these, local recurrence and distant metastasis occurred in 9 (21.4%) and 12 patients (28.6%), respectively. The 5-year OS, LRFS, and MFS rates for these patients were 81.1%, 78.2%, and 70.3%, respectively.

### 3.3. Prognostic Factor Analyses

**Figure 1:**
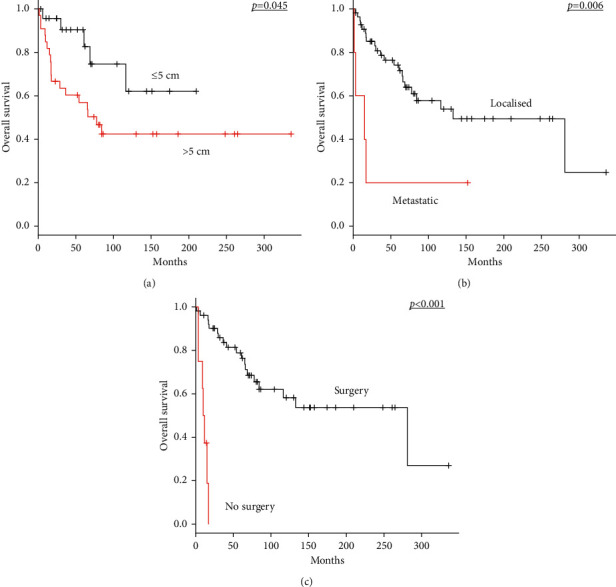
Kaplan–Meier survival curves in all 60 patients with MPNST. (a) OS according to tumour size (≤5 cm versus >5 cm). (b) OS according to stage (localised versus metastatic disease). (c) OS according to surgery of the primary tumour (presence versus absence of surgery). MPNST, malignant peripheral nerve sheath tumour; OS, overall survival.

For all 60 patients with MPNST, tumour size >5 cm (*p*=0.045), metastatic disease at initial diagnosis (*p*=0.006), and no surgery of the primary tumour (*p* < 0.001) were significant prognostic factors for unfavourable OS in univariate analyses (Table 1; Figures 1(a)−1(c)). The 5-year OS rate of patients without distant metastasis at initial diagnosis was 74.1%, whereas that of patients with initial metastasis was 20%. The median OS duration for patients with initial metastasis was 15 months (range, 1–152.5 months). The 5-year OS rate of patients who received surgery for the primary tumour was 78.9%, whereas that of patients with unresectable diseases was 0%. The median OS period for patients with inoperable tumours was 10.7 months (range, 3.3–16.8 months).

Multivariate analysis revealed that primary tumour surgery (hazard ratio (HR) 24.66; 95% confidence interval (CI) 4.972−122.3; *p* < 0.001) was the most significant prognostic factor for improved OS in all patients (Table 2).

**Figure 2:**
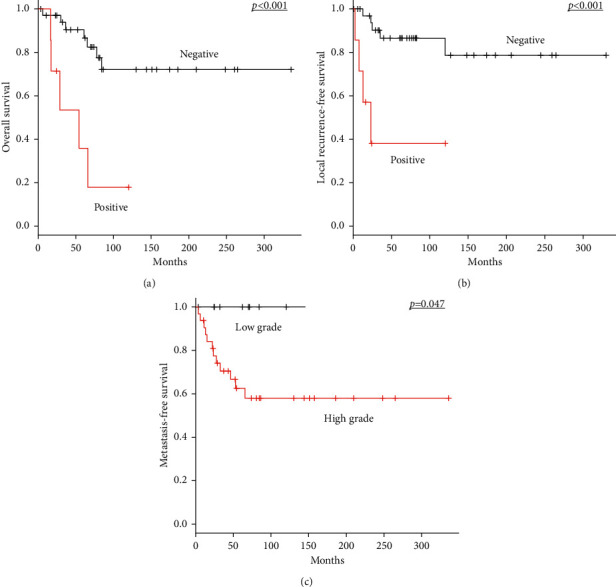
Kaplan–Meier survival curves in 42 patients with nonmetastatic MPNST who underwent surgery of the primary tumour at our institutions. (a) OS according to surgical margin status (negative margin versus positive margin). (b) LRFS according to surgical margin status (negative versus positive margin). (c) MFS according to histological grade (low grade versus high grade). MPNST, malignant peripheral nerve sheath tumour; OS, overall survival; LRFS, local recurrence-free survival; MFS, metastasis-free survival.

For 42 patients who had no distant metastasis at diagnosis and who underwent surgery of the primary tumour at our hospitals, a positive surgical margin was significantly associated with poor prognosis for OS (*p* < 0.001) and LRFS (*p* < 0.001) in univariate analysis (Table 3; Figures 2(a) and 2(b)). Moreover, patients with high-grade tumours significantly exhibited a poorer prognosis for MFS compared with those with low-grade tumours (*p*=0.047, Figure 2(c)).

**Figure 3:**
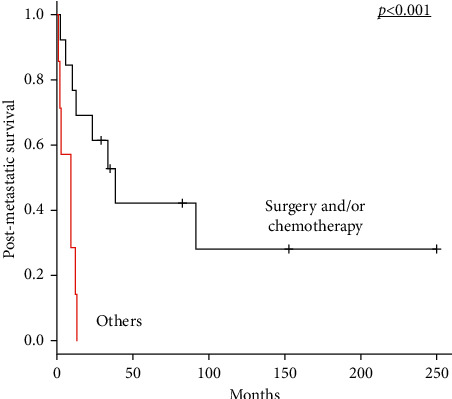
Kaplan–Meier survival curve of postmetastatic survival according to surgery and/or chemotherapy for metastatic lesions in 20 patients with metastatic MPNST who underwent surgery for their primary tumour (presence versus absence of surgery and/or chemotherapy for metastatic lesions). MPNST, malignant peripheral nerve sheath tumour.

Among the 52 patients who underwent surgical removal of the primary tumour, 2 had distant metastasis at initial diagnosis, and 18 developed distant metastasis during follow-up; their 5-year postmetastatic survival rate was 27.4%. As shown in Table 4, the number of metastatic lesions at diagnosis of metastasis was ≤3 in 14 patients. Among them, 7 patients underwent surgical resection of metastatic lesions, such as the lungs and lymph nodes. Systemic chemotherapy after the development of metastasis was performed in 10 patients. The number of metastases was not a significant prognostic factor for postmetastatic survival (*p*=0.161). As presented in Figure 3, the 5-year postmetastatic survival rate in 13 patients who were treated with surgery and/or chemotherapy for metastatic lesions, which was 42.2%, was significantly higher than that in seven patients who were not, which was 0% (*p* < 0.001).

## 4. Discussion

In patients with MPNST, the prognosis has remained poor, with 5-year survival rates ranging from 30% to 60% [3, 9, 12–23]. lThe reported long-term outcomes vary widely across published studies. The notable difference in our study from prior research is the higher OS. The 5-year OS rates of 69.5% among all patients and 81.1% among patients with localised disease at diagnosis who underwent surgery of the primary tumour at our institutions are higher than the rates previously reported in other studies. Several factors, such as metastatic disease; tumour grade, size, location, and surgical margin status; and NF1, have been indicated as significant prognostic predictors [9, 12–20, 22, 23, 25, 26].

Our study aimed to determine the factors affecting the clinical outcome in patients with MPNST who were treated at our institutions. Large-sized tumour, metastasis at diagnosis, and not having surgery of the primary tumour significantly predicted poor OS among all patients in the univariate analysis. Previous studies have emphasised the importance of surgery in MPNST treatment [13, 16]. Surgical resection of the primary tumour was also found to be the most significant independent prognostic factor for favourable OS in the multivariate analysis.

Among the 42 patients who had nonmetastatic diseases at the time of diagnosis who underwent surgery of the primary tumour at our institutions, local recurrence developed in 9 (21.4%). The local tumour recurrence in the present study was comparable with other studies, where values between 20% and 65% have been described [9, 12–14, 16–19, 21, 25–29].

Previous studies have shown that the rate of negative margins was 46.1%–87.9% [9, 14, 16, 18, 22, 23, 25]. Thirty-five patients (80%) who underwent resection with negative margins had better OS and LRFS outcomes in the current study. We believe that the survival outcomes reported in the present study are probably related to the relatively high rate of negative margins. However, complete surgical excision with negative margins is not always feasible due to tumour location or size. Several authors have recommended adjuvant radiotherapy to prevent local recurrence, but others have reported that radiotherapy was ineffective [8, 13, 14, 30].

All studies were based on retrospective settings, as no prospective randomised trials that specifically examine radiotherapy in the context of MPNST have been conducted. In our study, improvement in the rates of local control was not observed with adjuvant radiotherapy. The rate of adjuvant radiotherapy use in our study was lower than that in previous studies, probably due to the low rate of positive margins. Our results indicate that the mainstay of therapy for MPNST is surgical resection, with the goal of achieving complete removal with negative margins, and that the addition of radiotherapy following surgery may be considered in the adjuvant setting, especially when the surgical margins are positive due to the deep location of the tumour or its large size.

Despite the curative intent of treatment in localised MPNST, survival remains poor due to high metastatic potential. In the present study, among 42 patients with localised disease at diagnosis who underwent surgery of the primary tumour at our institutions, 12 (28.6%) developed distant metastases, mainly in the lungs. Tumour grade was described as a significant prognostic factor for MFS. In general, high-grade tumours have a greater propensity to metastasise and may, in principle, have a greater chance of benefiting from chemotherapy. MPNST appears to have intermediate chemosensitivity to various chemotherapeutic regimens adopted over the years, with response rates ranging from 21% to 45% [8, 15, 31–33]. In the present study, DXR/IFM showed an ORR of 27.3% and a DCR of 81.8%.

The use of neoadjuvant and/or adjuvant chemotherapy for MPNST has been debated. Several studies have failed to demonstrate a survival benefit for chemotherapy in MPNST treatment [9, 12, 19]. However, most of these studies were small and retrospective, encompassing patients treated with different regimens and often pooling data from multiple trials at multiple institutions. The use of neoadjuvant and/or adjuvant chemotherapy was also not significantly associated with an improvement in survival in our study.

Recent studies have demonstrated that neoadjuvant and/or adjuvant chemotherapy may be considered in high-grade, large, and deep MPNST [15, 31, 32, 34]. In the present study, neoadjuvant and/or adjuvant chemotherapy also tended to be administered more often to tumours exhibiting aggressive features. Our results indicate that neoadjuvant and/or adjuvant chemotherapy may not consistently provide a significant survival benefit for patients with MPNST but should be performed in patients with tumours with higher grade, larger size, and deeper location. Additional research is required to identify predictive biomarkers for therapeutic response to improve outcomes for patients with MPNST.

The survival rate of patients with metastatic MPNST is extremely poor, and thus, the disease remains difficult to manage. In previous reports, more than 10% of patients with MPNST present with unresectable or metastatic disease [15, 16, 19]. In addition, 20%–65% of patients receiving treatment with curative intent will develop metastatic disease [9, 12, 14, 17–19, 21, 25–28, 35]. In the present study, 16.7% of all patients presented with unresectable or metastatic disease, and 28.6% of those with localised disease receiving surgery of the primary tumour with curative intent at our institutions developed metastatic disease during follow-up.

Previous studies have shown 5-year survival rates in patients with metastatic MPNST ranging from 0% to 25% [9, 12, 14, 18, 21, 29]. However, prognostic factors for survival in those patients remain unclear. Complete resection of metastases has been considered as an important determinant of outcomes. The literature has demonstrated that selected patients with MPNST could benefit from resection of pulmonary metastases in particular [36, 37]. Complete resection of the metastatic lesions in the lungs and lymph nodes should be the most effective treatment to achieve long-term survival or even to cure selected patients.

In patients who are not eligible for resection of metastases, systemic chemotherapy has been employed in palliation to improve their quality of life by reducing the symptoms. Cytotoxic chemotherapy comprising anthracycline-containing regimens has long been the mainstay of treatment for unresectable and/or metastatic soft tissue sarcomas, including MPNST [34]. In the current study, among patients with MPNST with localised disease at diagnosis who received surgical resection of the primary tumour but developed distant metastases, one had a complete response to chemotherapy, indicating that chemotherapy can be significantly beneficial for selected patients. Moreover, four patients who were treated with surgery and/or systemic chemotherapy for metastatic lesions survived for more than 5 years after the development of distant metastases. The 5-year postmetastatic survival rate of patients who received surgery and/or chemotherapy for metastatic lesions was 42.2%, whereas that of patients who did not was 0%. The available treatment options for patients with metastatic MPNST are limited. However, our results indicate that, even if distant metastases occur, these treatments are essential for favourable outcomes and can likely result in prolonged survival in selected patients.

MPNST is the leading cause of death in patients with NF1 as these patients were reported to have an 8%–13% lifetime risk of developing MPNST [3, 11, 12]. Some studies have demonstrated that patients with NF1-related tumours have a worse OS than those with sporadic tumours [14, 15, 20, 23]. The larger size and deeper location of the tumour and more frequent truncal location of NF1-related MPNST have accounted for poor outcomes. Conversely, other studies have failed to demonstrate a reduction in survival [9, 12, 13, 16, 18, 19, 21, 22, 25].

The outcome differences between NF1-related and sporadic MPNST remain controversial due to the existence of conflicting data. In our analysis of 60 patients, we found no significant difference in survival between those with sporadic tumours and those with NF1-related tumours. Patients without NF1 seemed to present at an early stage, whereas patients with NF1 tended to present late, as they likely failed to recognise a malignancy early among the benign tumours they already had and developed over the years. Therefore, to discover MPNST as early as possible, patients with NF1 should be followed up carefully, given the likelihood that they will develop MPNST.

The present study has several limitations, such as its retrospective nature and the small number of patients. Thus, definitive conclusions could not be drawn. We were not able to obtain data on tumour size and grade as well as surgical margin in three cases or tumour depth in one case. Patients were not randomised to receive chemotherapy or radiotherapy, and administration regimens were not uniform. Selection bias regarding receipt of neoadjuvant and/or adjuvant chemotherapy and radiotherapy was possible. We tended to perform neoadjuvant and/or adjuvant chemotherapy on high-risk patients and failed to evaluate the effects of each appropriately.

## 5. Conclusions

The 5-year OS rate of all patients with MPNST was 69.5%. In the multivariate analysis, surgery of the primary tumour was significantly associated with favourable OS. The 5-year OS, LRFS, and MFS rates of patients with localised disease at diagnosis who underwent surgery of their primary tumour at our institutions were 81.1%, 78.2%, and 70.3%, respectively. Negative surgical margin was significantly associated with better OS and LRFS, and patients with high-grade tumours exhibited more unfavourable MFS.

Surgery and/or systemic chemotherapy for metastatic lesions could increase the survival of patients with distant metastases who underwent surgical resection of the primary tumour. Complete surgical excision of the primary tumour with negative margins remains the only proven curative treatment. We recommend a multidisciplinary treatment for patients with MPNST with aggressive features to maximise a good prognosis.

## Figures and Tables

**Table 1:** Patient-, tumour-, and treatment-related characteristics and univariate analysis of prognostic factors for OS in 60 patients with MPNST.

Factors		N (%)	5-year OS (%)	*p* value
Age	≤50	27 (45)	59.6	0.598
	>50	33 (55)	77.6	

Gender	Male	31 (51.7)	60.3	0.059
	Female	29 (48.3)	79.7	

NF1 status	Present	32 (53.3)	63.4	0.342
	Absent	28 (46.7)	76.8	

Location	Extremity	19 (31.7)	89.5	0.164
	Trunk	35 (58.3)	56.8	
	Head and neck	6 (10)	83.3	

Size	≤5 cm	24 (42.1)	90.3	0.045
	>5 cm	33 (57.9)	57	
	NA	3	—	—

Depth	Superficial	20 (33.9)	82.5	0.367
	Deep	39 (66.1)	62.6	
	NA	1	—	—

Grade	1	12 (21.1)	100	0.095
	2 or 3	45 (78.9)	62.6	
	NA	3	—	—

Stage	Localised	55 (91.7)	74.1	0.006
	Metastatic	5 (8.3)	20	

Surgery	Yes	52 (86.7)	78.9	<0.001
	No	8 (13.3)	0	

OS, overall survival; MPNST, malignant peripheral nerve sheath tumour; NF1, neurofibromatosis Type 1; NA, not available.

**Table 2:** Multivariate analysis of prognostic factors for OS in 60 patients with MPNST.

Factors	HR	95% CI	*p* value
Size >5 cm	1.961	0.7–5.491	0.2
	1		

Metastasis at diagnosis	0.751	0.182–3.105	0.693
	1		

No surgery	24.66	4.972–122.3	<0.001
	1		

OS, overall survival; MPNST, malignant peripheral nerve sheath tumour; HR, hazard ratio; CI, confidence interval.

**Table 3:** Patient-, tumour-, and treatment-related characteristics and univariate analysis of prognostic factors for OS, LRFS, and MFS in 42 patients with nonmetastatic MPNST who underwent surgery of the primary tumour at our institutions.

Factors		*N* (%)	5-year OS (%)	*p* value	5-year LRFS (%)	*p* value	5-year MFS (%)	*p* value
Age	≤50	20 (47.6)	71.7	0.522	83.9	0.294	72.1	0.665
	>50	22 (52.4)	90		73.2		68.3	

Gender	Male	18 (42.9)	76	0.34	76.7	0.981	58	0.26
	Female	24 (57.1)	84.8		80.3		80.7	

NF1 status	Present	21 (50)	79.1	0.531	73.7	0.73	58.6	0.067
	Absent	21 (50)	83.1		83.6		83.6	

Location	Extremity	17 (40.5)	94.1	0.896	78.7	0.913	68.4	0.705
	Trunk	21 (50)	73.2		78.5		72.8	
	Head and neck	4 (9.5)	75		75		75	

Size	≤5 cm	19 (45.2)	87.7	0.389	87.5	0.576	82	0.233
	>5 cm	23 (54.8)	77.3		72.1		63.4	

Depth	Superficial	16 (38.1)	84.4	0.644	80.8	0.836	71.4	0.992
	Deep	26 (61.9)	79.5		75.5		70.4	

Grade	1	10 (23.8)	100	0.07	100	0.095	100	0.047
	2 or 3	32 (76.2)	76.4		72.1		62.5	

Margin status	Negative	35 (80)	90.5	<0.001	86.5	<0.001	74	0.229
	Positive	7 (20)	35.7		38.1		47.6	

N/A CT	Yes	11 (26.2)	78.7	0.692	80.8	0.621	77.9	0.818
	No	31 (73.8)	82.1		76.9		67.3	

N/A RT	Yes	8 (19)	75	0.691	75	0.851	70	0.715
	No	34 (81)	82.6		78.4		69.9	

OS, overall survival; LRFS, local recurrence-free survival; MFS, metastasis-free survival; MPNST, malignant peripheral nerve sheath tumour; NF1, neurofibromatosis Type 1; N/A CT, neoadjuvant and/or adjuvant chemotherapy; N/A RT, neoadjuvant and/or adjuvant radiotherapy.

**Table 4:** Patient-, tumour-, and treatment-related characteristics and univariate analysis of prognostic factors for postmetastatic survival in 20 patients with metastatic MPNST who underwent surgery of the primary tumour.

Factors		*N* (%)	5-year postmetastatic survival (%)	*p* value
Age	≤50	6 (30)	50	0.444
	>50	14 (70)	17.9	

Gender	Male	14 (70)	28.6	0.855
	Female	6 (30)	25	

NF1 status	Present	12 (60)	27.8	0.666
	Absent	8 (40)	25	

Location	Extremity	8 (40)	31.2	0.48
	Trunk	9 (45)	22.2	
	Head and neck	3 (15)	33.3	

Size	≤5 cm	5 (29.4)	20	0.693
	>5 cm	12 (70.6)	31.2	
	NA	3	—	—

Depth	Superficial	6 (31.6)	0	0.384
	Deep	13 (68.4)	38.5	
	NA	1	—	—

Margin status	Negative	10 (50)	33.3	0.149
	Positive	10 (50)	20	

Number of metastases	≤3	14 (70)	34.3	0.161
	>3	6 (30)	0	

Surgery for metastases	Yes	7 (35)	53.6	0.059
	No	13 (65)	11.5	

Chemotherapy for metastases	Yes	10 (50)	25	0.366
	No	10 (50)	30	

Surgery and/or chemotherapy for metastases	Yes	13 (65)	42.2	<0.001
	No	7 (35)	0	

MPNST, malignant peripheral nerve sheath tumour; NF1, neurofibromatosis Type 1; NA, not available.

## Data Availability

The datasets used in this study are available from the corresponding author upon reasonable request.
